# PrEP your step: Implementing an online crowdsourcing contest to engage young people in HIV prevention in Washington DC, USA

**DOI:** 10.1371/journal.pone.0313882

**Published:** 2024-11-18

**Authors:** Tamara Taggart, Allison Mathews, Toni Junious, Joseph A. Lindsey, Andrea Augustine, Charles Debnam, Yavonne Boyd, Seraiya Wright, Joseph D. Tucker, Manya Magnus

**Affiliations:** 1 Department of Prevention and Community Health, George Washington University, Washington, DC, United States of America; 2 Department of Social and Behavioral Sciences, Yale University, New Haven, Connecticut, United States of America; 3 Community Expert Solutions, Inc., Winston-Salem, North Carolina, United States of America; 4 Planned Parenthood of Metropolitan Washington, DC, Washington, DC, United States of America; 5 Community Wellness Alliance (CWA), Washington, DC, United States of America; 6 Mary’s Center, Washington, DC, United States of America; 7 Institute for Global Health and Infectious Diseases, University of North Carolina at Chapel Hill, Chapel Hill, North Carolina, United States of America; 8 Clinical Research Department, Faculty of Infectious and Tropical Diseases, London School of Hygiene and Tropical Medicine, London, United Kingdom; 9 Department of Epidemiology, George Washington University, Washington, DC, United States of America; University of California San Diego, UNITED STATES OF AMERICA

## Abstract

HIV incidence among young people (Black and Latinx women and men who have sex with men ages 16–24 years), in the United States is high. Traditional top-down approaches for pre-exposure prophylaxis (PrEP) social marketing are not effectively reaching this population. Crowdsourcing is a promising approach to engaging young people in the development of innovative solutions to raise awareness and use of PrEP among those at highest risk of HIV. This study engaged young people in the design and evaluation of an online crowdsourcing contest to promote PrEP among Washington, DC youth. The contest used standard methods recommended by the World Health Organization and feedback from our community partners. Online recruitment using social media elicited online votes and survey responses. We analyzed cross-sectional surveys using descriptive statistics, and semi-structured interviews with contest participants using thematic coding to explore barriers and facilitators to contest engagement. Approximately 82% of entries were from young people in DC. A convenience sample of 181 people voted on their favorite crowdsourced PrEP messages and shared their awareness and attitudes about PrEP. The contest website received 2,500 unique visitors and 4,600 page views. Themes from semi-structured interviews (n = 16) included the need for more community engagement in developing PrEP messaging and positive attitudes towards crowdsourcing. Survey data (n = 887) showed that the crowdsourced messages were well-liked and resonated with the community. Most preferred to see PrEP messages in social media (23%), email (17%) and videos (14%). Approximately 70% of survey participants reported that after viewing the crowdsourced message they would talk to their sexual partner or medical provider (63%) about PrEP, use PrEP (58%), and learn more about PrEP (56%). Crowdsourced messages solicit substantial online viewership. More implementation research is needed to understand the public health impact of integrating social media, crowdsourcing, and community engagement to develop PrEP promotional messages.

## Introduction

There is a critical need to reduce HIV incidence among young people (ages 16 to 24 years). Within the United States (U.S.), HIV rates among key groups of young racial/ethnic minorities (Black and Latinx men who have sex with men (MSM) and cisgender women) continue to increase. In 2020, youth aged 24 years and younger accounted for an estimated 20% of all new HIV diagnoses [1]. In Washington, DC (D.C.), Black women and racial/ethnic minority MSM represent 1 in 5 and 2 in 5 new HIV infections, respectively, with the majority of these infections occurring among young people [2]. Persistent HIV inequities suggest that current HIV prevention strategies are not meeting the needs of the HIV epidemic within these populations. Combination behavioral and biomedical (e.g., pre-exposure prophylaxis) HIV prevention strategies have the potential to address disparate rates of HIV [3].

Pre-exposure prophylaxis (PrEP) is a proven effective biomedical intervention for the prevention of HIV. When taken as prescribed, PrEP prevents HIV acquisition by more than 90% and is well tolerated by young people [4–6]. PrEP has been approved by the U.S. Food and Drug Administration (FDA) for the prevention of HIV in adults since 2012. This indication was extended to people under 18 years in 2018, and most recently, clinical guidelines for PrEP have expanded to include all individuals who are sexually active [7]. Despite this public health advancement, awareness and use of PrEP among key groups of young people remains low [8, 9] For example, in 2018, people ages 13–25 years had the lowest rate of PrEP uptake of any age group other than those older than 55 years [10]. Moreover, only 5.6% of all PrEP users are women of which, only 26% are Black [11]. Barriers to uptake and wider use of PrEP among key groups of young people in the U.S. include a complex array of social-structural and behavioral factors including knowledge and awareness of PrEP, stigma, fear, PrEP costs, and access to PrEP [3, 12–14]. PrEP stigma, fear, and lack of awareness about PrEP are barriers that may be mitigated by using social media and community-engaged approaches to ensure that messaging about PrEP is developmentally relevant, resonates, and does not further stigmatize the intended populations of interest [15].

One community engagement approach that has shown some effectiveness in developing community-supported messaging about PrEP is crowdsourcing. Crowdsourcing has a group of people solve all or part of a problem, then share back selected solutions with the public [16, 17]. Crowdsourcing engages a broad range of people, including members of key populations, creatives, and community leaders to develop solutions to an innovation challenge. Compared with traditional approaches to message development that may use only one or limited community engagement strategies, open contests (a form of crowdsourcing), that use multiple engagement strategies including various social media and networking sites and in-person engagement have a greater potential to reflect local culture and social context g enhance the relevance, fit, and quality of PrEP messages [18]. Crowdsourcing has been used in the public health sector to promote HIV testing and prevention, smoking cessation, vaccination, and public health policy [18–24]. The strengths of crowdsourcing are numerous, including the opportunity for participation from people who may be less motivated to engage in ongoing participatory activities like stakeholder meetings, the ability to quickly gather a range of diverse perspectives, and a greater potential for innovation and relevance as compared to more traditional approaches [18, 22, 24, 25]. Crowdsourcing is also strengthened by the use of technology and social media, which enhances accessibility, anonymity, and tailoring information to individual needs [15].

Current PrEP messages fail to engage Black and Latinx MSM and women and PrEP uptake remains low for those who demonstrate the most need [9]. PrEP promotion continues to perplex public health officials suggesting a need for new approaches to message development. Few PrEP messaging campaigns have been designed with and evaluated by young racial/ethnic and sexual minorities. Lastly, few have leveraged the strengths of social media to facilitate message design with the communities who demonstrate the most need [15]. Leveraging technology and social media with community-engaged strategies has the potential to mitigate these challenges and address persistent disparities in PrEP awareness and use. The specific objectives of the study were to: 1) implement an open contest to capture community responses to a public health challenge—developing messages to promote PrEP awareness among key groups of young people, and 2) determine the likeability and relevance of crowdsourced PrEP messages to this group.

## Materials and methods

The data for this study were drawn from cross-sectional surveys and semi-structured interviews conducted between 01 December 2019 and 01 March 2022. We conducted formative research (i.e., systematic review of social media campaigns to increase PrEP awareness and uptake, and key informant interviews with healthcare professionals including PrEP providers and HIV specialists) to inform our contest design and gain more insights into PrEP provisions in D.C. [15] Our methods were guided by the principles of community-based participatory research (CBPR) which prioritizes active involvement of community members, organizations, and researchers in all aspects of the research process to enhance understanding of a phenomenon and develop action-oriented solutions to benefit the community involved [26] We also formed a youth steering committee which consisted of eight Black and Latinx youth ages 16–22 years. Our steering committee was selected based on their participation in local community-based organizations that support sexual health and youth empowerment. Youth were interviewed, invited to serve on the steering committee, and committed to participating in up to six meetings a year; steering committee members were compensated for their time and expertise. This group was representative of our focus population and participated in feedback sessions to help shape details of the contest, including the contest design, prompt (enter one or two sentences or a #hashtag on “what you could do or say to spread the word about PrEP”), criteria for contest submissions, and development of recruitment strategies. We modified and used a WHO practical guide to inform the development of the crowdsourcing contest (see Table 1 for a description of the stages used in this study) [17, 27].

**Table 1:** Key stages of using crowdsourcing to promote public health through open contests and designathons.

	Overview	Structure
**Open Call**	**Organizing a call for entries**	A diverse steering committee of community members and researchers establishes an open call for entries including contest rules and methods for engagement.
	**Engaging community to contribute**	Social media and in-person events at community organizations, schools, local radio, and other local venues frequented by the focus population.
	**Evaluating open call entries**	Steering committee and other community representatives evaluate entries based on pre-specified criteria. Crowd votes on top scoring entries.
	**Recognizing finalists with prizes**	Social media recognition of finalists including written and video posts; prizes awarded.
**Designathon**	**Using finalist content to create community engagement strategies**	Finalist content shared with community stakeholders, presented in public health settings, and disseminated in other spaces to facilitate developing PrEP messages.

The study was open to all people ages 16 years and older, however recruitment efforts focused on reaching young Black and Latinx women and MSM ages 16–29 years living in the Washington, DC metropolitan area. We implemented geofencing recruitment strategies (e.g., paid promotion of Facebook, Instagram, and Twitter posts, and promotion of ads on sexual networking platforms like Jack’d and Grindr); advertising with local radio stations that catered to predominantly Black or Latinx listeners; and recruitment emails to community partners, clinicians, and local student organizations. We also attended community events and posted flyers in locations frequented by the focus population (e.g., local teen-only nights, sporting events, and college campuses), which allowed study staff to engage the community and answer questions about the research study and contest participation.

**Fig 1:**
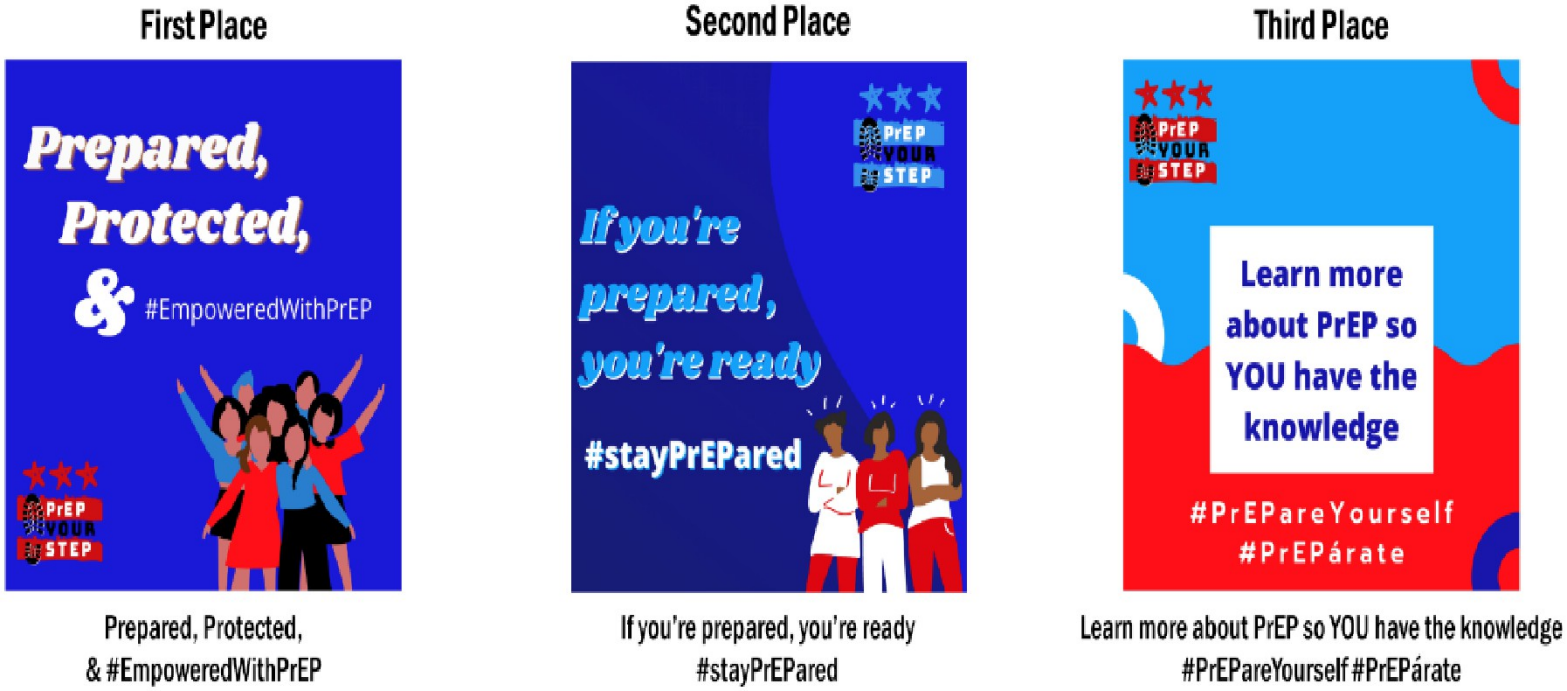
Top three crowdsourced messages.

Online contest submissions were captured through the study website (PrEPyourstep.org), which also provided information about the study and local HIV prevention resources. Participants submitted responses to the contest prompt through the website. Responses were collected for four months. Two members of the research team screened all submissions for duplicates, completeness, and relevance to the scope of the contest. Next, two members of the research team independently rated the submissions with scoring criteria used in other crowdsourcing projects and feedback from the youth steering committee [20, 22, 25]. Specifically, submissions were rated across seven items on a scale of 1 (least) to 10 (most). Scoring criteria included relevance (i.e., is the message appropriate for the focus population?; does the message consist of language that represents the target population?), accuracy (i.e., is the information in the message correct?), impactful (i.e., will the message significantly influence PrEP awareness and use among the focus population?), and other factors related to creativity and message appeal (i.e., can the message be viewed by various audiences and have the same meaning?, how creative and innovative is the message?, will youth feel comfortable (not embarrassed) sharing this slogan or hashtag with their friends?). The top eight entries were sent to a panel of expert judges (n = 6, PrEP providers, health communication experts, public health officials, and staff from community-based organizations that support HIV prevention among young people) and the youth steering committee for scoring using the same criteria. The five entries with the highest scores were posted on our social media for four weeks for public voting. The finalist messages are shown in Fig 1. Prize incentives included a $350 gift card (first place), a $250 gift card (second place), and a $150 gift card (third place).

We randomly selected contest participants to complete a semi-structured interview with a trained research assistant via video conferencing. The semi-structured interview guide focused on motivations for participating in the contest, PrEP awareness, and contest impact on HIV prevention engagement. The interview guide was developed in consultation with the study youth steering committee. Sample questions included, “What made it easy for you to participate in the contest?” and “Before the participating in the contest, had you heard of PrEP? If so, what did you know? How did you hear about PrEP?”. Interviews were on average 33.5 minutes and were recorded, transcribed, reviewed, and coded using Dedoose web-based qualitative software. Coding was inductive and included descriptive and conceptual codes. Codes were reviewed and developed into key themes by the first author in discussion with the research team. Themes and representative quotes were shared with the youth steering committee and community partners for feedback and further refinement.

We administered a brief message testing survey online using Qualtrics software. The survey consisted of 43 items and assessed sociodemographic characteristics, PrEP awareness and use, and features of the crowdsourced messages including message likability, persuasiveness, and channels. Survey participants were recruited through social media and were entered into a raffle to receive one of 10 gift cards. Online engagement measures were extracted using Facebook and Instagram analytics for the study’s social media sites and Google Analytics for the study website. Measures included page follows (unique users who subscribe to page update alerts) and page visits (unique users who visited a page). Summary statistics were used to describe message testing survey responses and online engagement metrics. Participants provided online consent and assent for all aspects of the study. All study activities were reviewed and approved by the George Washington University Institutional Review Board (#NCR191582) prior to study initiation.

## Results

There were 67 contest entries from 67 contestants. Of those who submitted an entry, 82% (n = 55) were members of the focus population (Black and Latinx MSM and women ages 16–29 years), 69% (n = 46) were aware of PrEP prior to participating in the contest, 24% (n = 16) were currently using PrEP, and 63% (n = 42) reported ever receiving an HIV test. All 67 entries were scored using the contest rubric and scores ranged from 1 to 62 points out of a total of 63 possible points. In total, 181 people voted on the top five entries and the top three finalists were announced on our social media (see Fig 1). PrEPyourstep.org, the study website, received 2,500 unique visitors and 4,600 page views during the study period. Our social media received 330 page follows, which included a mix of members of the focus population, community-based organizations, social media creatives and personalities in the D.C. metropolitan area, and HIV advocates.

### Post-contest interviews

We conducted 16 semi-structured interviews, a number determined by reaching data saturation in which no additional insights emerged from interviews, signifying an adequate sample size for data analysis. Interviews were conducted with contest participants after contest winners were announced. Interview participants were representative of our focus population. All participants were racial/ethnic minorities (i.e., Black and Latinx). Most were between the ages of 16 and 29 years (n = 15, 94%), female (n = 9, 56%), and a sexual minority (n = 8, 50%). Key themes that emerged from the interviews included: 1) developing PrEP messages through open contests is appealing to the community, 2) motivations for contest participation, and 3) impact of contest participation on attitudes towards PrEP and behaviors.

#### Developing PrEP messages through open contests is appealing to the community

Many participants shared the aspects of the study that made it interesting (i.e., appealed) to them. These factors included the design of the contest and the overall approach. Participants expressed their initial interest was driven by the opportunity to have their voices heard and be part of efforts to develop PrEP messages for future use. As one participant stated, “I think if you continue to give people the opportunity to have a say in the research and what will be the work as a product. You keep giving the community an opportunity to be involved in that. Also, representing more of the community that I am a part of. Then, I feel great.” Other participants noted that the open contest strategy appealed to them because of the focus on hearing from the community and the use of social media to facilitate community input. As one participant shared, “the community engagement and collaboration were just so innovative to me.” While another participant shared that participating in the contest during the COVID-19 pandemic made them more aware of how social media can be leveraged to engage communities in public health. Stating that he was, “definitely encouraged to be creative with how we encourage other people to take care of their health.”

#### Motivations for contest participation

Participants described their motivations (e.g., factors that encouraged or prompted them to participate in the study) for either submitting a contest entry, voting on their favorite entries, or both. Most participated because they liked the opportunity to be creative and felt empowered by the premise of the contest, which was for communities to lead the development of PrEP promotion messages. One participant shared, “I think that it’s good to get new perspectives and new ideas out on how to encourage people to take more charge of their own sexuality.” While another shared, “I feel good to know that I’m contributing to something that will help the youth make more educated decisions around their sexual health and well-being.” These two participants echoed what many said motivated them to participate, including the ability to share new ideas and innovations about PrEP messaging based on their knowledge of HIV and HIV prevention, contribute to something that will help young people, and be connected to something in their community that will serve a greater good. Additionally, participants commented that the ease of participation motivated them. As one participant shared, “What made it easy was how accessible it was. Electronically, I can get to it [study website and social media]. It is very user friendly. Everything laid out in the instructions. It was easy to follow.”

#### Impact of contest participation on PrEP attitudes and behaviors

Participants shared that participating in the contest increased their knowledge about PrEP and HIV prevention and improved their attitudes towards PrEP. One participant shared that the contest, “made me think about the decisions I make when it comes to sex, unprotected sex, and using PrEP.” While another participant shared, “The biggest lesson I’ve learned from participating would be that talking about HIV is normal…Things like the whole campaign around it shows that it doesn’t have to be a bad thing. We can talk about it. People still live normal lives, and now there’s another way to prevent it.” Other participants echoed these comments by saying that their participation in the contest helped them understand how PrEP is “normal” and “empowering” for young people, especially those who may identify as sexual minorities. Although none of the participants interviewed reported increased PrEP uptake as a result of participating in the contest, most remarked on how their participation improved their attitudes toward and interest in PrEP and HIV prevention.

### Message testing survey

In total, 887 people completed the message testing survey. Approximately 73% (n = 647) of the respondents were from the focus population (Black and Latinx women and MSM ages 16–29 years who live in Washington, DC). Most were employed full- or part-time (55%, n = 485); 27% (n = 236) had a high school diploma, 31% (n = 271) completed some college, and 22% (n = 195) had a bachelor’s degree. Approximately 89% (n = 793) had heard of PrEP prior to viewing the crowdsourced messages, 47% (n = 418) had ever taken PrEP, and 72% (n = 63) had ever taken an HIV test. Most participants reported that they preferred to see messages about PrEP promotion on social media (n = 208, 23%) including Facebook, Snapchat, Instagram, and Twitter. Email (n = 155, 17%) and Internet video streaming services (n = 128, 14%) were the next preferred. Mobile sexual networking applications such as Grindr, Jack’d, and Tinder (n = 106, 12%), SMS text messages (n = 84, 9%), and print media (n = 81, 9%) were among the least preferred channels. Approximately 70% (n = 622) of participants reported that after viewing the crowdsourced messages they would talk to their sexual partner or medical provider (n = 561, 63%) about PrEP, use PrEP (n = 516, 58%), and be motivated to learn more about PrEP (n = 499, 56%). Most participants agreed that the crowdsourced messages made them think about PrEP (n = 482, 54%) and were appealing (n = 593, 67%). After viewing the crowdsourced messages, compared to MSM, women reported significantly higher intention to talk to their medical provider about PrEP (Mean[SD], MSM = 3.04[0.67] and Women = 3.50[0.69], p = .033). No significant differences between MSM and women were observed in interest in learning more about PrEP, message likability, or channel preference. Next we examined differences between those who had never taken PrEP and those who were PrEP experienced (defined as had ever taken PrEP). After viewing the crowdsourced messages, compared to individuals who have never taken PrEP, those with PrEP experience reported significantly higher interest in learning more about PrEP (Mean[SD], PrEP experienced = 3.17[0.77] and never taken PrEP = 3.10[0.59], p = .040) and message likeability (Mean[SD], PrEP experienced = 3.66[0.27] and never taken PrEP = 3.60[0.19], p = .018). No significant difference was observed in intention to talk to their medical provider about PrEP or channel preference.

## Discussion

The purpose of this study was to assess the feasibility and implementation of a crowdsourcing contest to develop PrEP promotion messages for young Black and Latinx MSM and women in Washington, DC. Using web-based analytics, qualitative interviews, and surveys, our study shows that crowdsourcing serves as promising and successful approach to integrating CBPR with digital technologies to engage young people in PrEP messaging. Our findings also underscore the importance of using community-engaged strategies to develop PrEP messaging to reflect community concerns and insights, amplify community voices, and utilize the guiding principles of health equity and community capacity building [28, 29].

Findings from the one-on-one interviews with contestants revealed themes that may be useful for future public health initiatives designed to raise awareness and uptake of PrEP among young people in D.C. The three interconnected themes include the appeal of including community voices in developing PrEP messages; excitement about the opportunity to contribute ideas to solving a public health challenge and the ease of online participation; and an improvement in favorable attitudes towards PrEP and HIV prevention. Similar to other research, our findings show that participants were enthusiastic, engaged, and highly motivated to contribute to the contest and learn more about HIV prevention among key groups of young people in the U.S. [20, 30, 31]. Moreover, contest engagement—measured through Facebook and Instagram analytics for the study’s social media sites and Google Analytics for the study website—revealed consistent engagement throughout the study. However, a limitation of this approach to measuring contest engagement is that we are unable to determine whether we reached the most vulnerable members of the focus population (e.g., those who meet clinical guidelines for PrEP, but are not taking PrEP), the duration of engagement on each virtual contest platform and throughout the contest, and the number of unique users across the different platforms (i.e., were the same participants accessing information on the study website and social media or did some participants only use one modality). Future research is needed to determine the best strategies for accurately measuring contest engagement and whether the modality used for engagement has an effect on an individual’s contest experience and subsequent behaviors.

Interview findings also revealed the importance of designing digital crowdsourcing contests that are community-driven, incorporate youth perspectives, and integrate in-person recruitment strategies with online activities. Similar to other studies that have integrated CBPR with crowdsourcing, our study actively sought community input at every stage of the contest, using various in-person and digital engagement strategies. These strategies are known to increase participation and the local relevance of identified solutions [19, 32]. Although the number of projects that integrate crowdsourcing with CBPR is increasing, these strategies remain underutilized in PrEP promotion. Our study contributes to the growing evidence that crowdsourcing may provide a unique and strengths-based approach to engaging communities in developing strategies to promote PrEP use. Overall, the entries received from the open contest are indicative of youth beliefs about PrEP, highlighting the significance of implementing broad engagement strategies to effectively reach this specific population. Public health initiatives designed to promote PrEP awareness and uptake among key groups of young people should prioritize community engagement through multiple and diverse settings and channels (e.g., in-person engagements in different settings, social media platforms, and other digital sources) [29]. This approach may lead to initiatives that resonate and are more effective in promoting PrEP usage among young people.

Findings from our message testing survey revealed that crowdsourced messages about PrEP were creative, appealing, and encouraged discussion about PrEP and HIV prevention with sexual partners, peers, and clinicians. Similar to findings from other studies on PrEP messaging, messages that de-stigmatize PrEP use and are easy to comprehend are preferred by young people [12, 33]. Survey findings also showed that while young people prefer to see messages about PrEP online, they are less interested in seeing them on sexual networking and dating applications. Differences in PrEP attitudes by gender, sexual orientation, and PrEP experience provide insights for future crowdsourcing campaigns with these groups. Collectively, these finding suggests the need to conduct research that examines the level of engagement in HIV prevention on web-based platforms, as well as how preferences for engagement may shift over time and context in different ways based on key sociodemographic factors [34].

There are several limitations to this study. We used a cross-sectional design, which limits our ability to understand how existing PrEP knowledge, access, and use may influence contest engagement and results. This limitation means that study results may be biased towards individuals who are already familiar with PrEP. Although our contest website and social media pages were widely available, study participation was restricted to individuals who resided in the D.C. metropolitan area. This region is known for its high HIV incidence and prevalence rates among key groups of young people. Thus, the themes and findings derived from this study are not generalizable to other communities among which HIV inequities persist. Additionally, our cross-sectional methods do not allow for repeated assessments nor did we randomize survey participants to receive crowdsourced and traditionally-created PrEP messages which limits our ability to assess message effectiveness and make meaningful comparisons between the two approaches. Lastly, although interest and favorable behaviors towards PrEP are noted proxies for PrEP use, we did not measure actual PrEP use or HIV-prevention engagement. More research is needed to address these limitations before implementing this approach to message development within public health efforts to increase PrEP use.

Despite the aforementioned limitations, findings from this study provide valuable insights that may inform future PrEP research and practice. It is imperative that future investigations include the development of advocacy for more youth-centered HIV prevention strategies and policies. By prioritizing the needs and perspectives of young people, these strategies can be better tailored to address the unique challenges faced by this population. There is also a need for more youth-focused and co-created tools that empower young people and support their ability to make informed choices about their sexual health, including PrEP. Study findings also emphasize the importance of developing messaging that empowers young people, challenges misconceptions about PrEP, and de-stigmatizes PrEP use. Lastly, integrating CBPR, crowdsourcing, and digital technologies may be a promising and innovative model for equitable engagement of community members in developing targeted PrEP promotion strategies and messages.
